# Impacts of inter-annual climate variability on reproductive phenology and postnatal development of morphological features of three sympatric bat species

**DOI:** 10.1038/s41598-023-35781-6

**Published:** 2023-05-29

**Authors:** Hojjat Eghbali, Mozafar Sharifi

**Affiliations:** Department of Biology, Faculty of Science, Razi University, Kermanshah, Iran

**Keywords:** Ecology, Ecology

## Abstract

Inter-annual variation in weather conditions has been shown to affect the reproductive phenological patterns of many organisms. Because of their relatively small body size and dependence on ectothermic prey, temperate-zone insectivorous bats are particularly sensitive to adverse spring environmental conditions that affect the duration of gestation and timing of parturition in these animals. This study aimed to compare phenological recruitment, birth seasonality and synchrony and morphological changes during postnatal growth in *Rhinolophus* *euryale*, *Rhinolophus* *ferrumequinum* and *Myotis* *emarginatus* in two consecutive years representing a typical dry (2015) and an extremely wet climatic event (2016) in a nursing colony in Kerend cave, western Iran. Females of these three bat species arrived from their wintering cave to the nursing colony in late April to mid-May each year. Synchrony of parturition as defined by amount clustering of births within a year assessed by circular statistics showed that for *R*. *euryale* and *R*. *ferrumequinum* the angular variance in dry year were significantly (P < 0.05) lower than in wet year, indicating a low level of synchrony in 2016. Similar comparison showed that births from *M*. *emarginatus* were highly synchrony, and there were no significant differences in timing of births among years (*P* > 0.05). Generalized estimating equation (GEE) for *R*. *euryale* indicated that for body mass and forearm length tests of parallelism (interaction term or growth rate) and tests for equal intercepts (y-intercepts or group term) were significant (P < 0.001). In *R*. *ferrumequinum*, the initial (y-intercepts) forearm length and body mass were not significantly (P > 0.05) different between the 2 years, but the tests for parallelism showed a significant decrease in growth rates of body mass and forearm length in the wet year (P < 0.05). Similar comparison in *M*. *emarginatus* indicated that for body mass, tests of parallelism were significantly different (P = 0.004), while tests for equal intercepts were not (P = 0.23). Our results suggest that climate changes may have unequal effects on different bat species due to differences in foraging habitat, niche partitioning, reproductive requirements and foraging strategies.

## Introduction

The available data on global climate over the past century indicate that the Earth is warming and in recent decades, interest in forecasting impacts of this phenomenon has received much attention to how organisms respond to changes in climatic conditions^1,2^. It is well documented that concurrent with increases in atmospheric CO_2_ and other greenhouse gases, the global average in land and ocean surface temperature has increased by approximately 0.85 °C (0.65 ± 1.06 °C)^3,4^. Depending on global region, the average is predicted to rise to 1.1–6.4 °C by 2100^5–7^. Along with the increase in ambient air temperature, rise in spatial and temporal rainfall regimes and increased variability at the climatic extremes are the major impacts of climate change^8,9^. However, due to change in climate, the frequency of rain has become unpredictable, which has caused floods in one part and drought in the other in most countries. Therefore, impacts of changes in pattern and magnitude of precipitation and temperature as well as an increase in extreme events have profound effect on food supply and bio-social conditions of ecosystem^10^.

Climate is a significant ecological driver affecting key aspects of the breeding biology of organisms, including their phenological recruitment, birth synchrony, growth and reproductive output^11,12^. Various natural climatic factors along with an environmental or disturbance gradient can cause individuals, populations or biotic communities to shift at certain ecological edges^13,14^. Rapid environmental and anthropogenic changes, including climate change, are restructuring ecological communities by changing species distribution, abundance and interactions^15^. There is mounting evidence which documents that the recent global changes in climate have caused a considerable spatial and temporal rearrangement of natural communities everywhere in the world^16–18^. Additionally, the impact of climate change has caused many changes in the seasonal timing of species life-history events^16,19^. Such changes in species-specific phenological responses can alter the timing of various species interaction^20–22^ which have important biotic interactions in formation of new communities and ecosystem processes^23–25^.

In periodic environments, seasonality in the timing of mammal reproduction events is common^26^. Moreover, the timing and synchrony of births are also key components of fitness among various taxa living in areas with seasonal environments^27^. In ecosystems with seasonal environments, such patterns of reproduction characterized by reproductive synchrony are the results of natural selection^28^. Typically, in these areas parturition in mammals is timed to coincide with climatic conditions favourable for survival of the offspring. In the context of the world, climate change has consistently proved that rainfall variation causes inter-annual variation in the timing of birth peaks and birth synchrony^29^. In studying the impact of climate on reproduction of terrestrial mammals, many authors have reported a correlation between the environmental conditions and reproduction of these populations. For example, Ogutu et al.^29^ have found that some species of terrestrial mammals performed a delayed parturition in dry years and advanced parturition in rainy years. Similarly, studies on the impact of climate change on northern large mammals have shown that climatic variability had constraining effects on the timing and synchrony of births in some species, including *Ovis* *canadensis*^30^, *Rangifer* *tarandus*^12,31,32^, *Ovis* *dalli*^33,34^ and *Capreolus* *capreolus*^35^.

Most investigations aiming to appreciate the effect of climate on bats have been conducted in northern hemisphere. The results of these studies suggest that in rainy and cool springs a delay in the delivery period or in birth synchrony is usually expected to occur^36–40^. Some studies have shown that the timing of reproduction events in bats is strongly dictated by climatic factors such as ambient temperature or level of precipitation as these two factors regulate food availability for insectivore bats^41–43^. Sherwin et al.^42^ suggested that the biogeography, foraging and roosting behavior, reproduction and development, frequency and duration of torpor and hibernation of bats are changing in response to a changing climate. The cause of such changes in reproductive asynchrony in parturition is believed to be an intense slowdown of gestation that follows when bats enter torpor in a rainy period in spring^44–46^. It is not clear that the influence of reduction in ambient temperature or increase in precipitation on the delay in parturition or reduction in birth synchrony is direct or indirect, since bats are assumed to enter torpor when the weather is appropriate^47^. Early laboratory studies aiming to categorize various climate factors on bat reproduction showed that both temperature and precipitation factors can interact^48^. However, some studies have shown that spring precipitation and low temperatures are often positively correlated with length of pregnancy in bats^11,44,49,50^.

In temperate regions, variation in spring temperature is known to affect various aspects of reproduction including initiation^51^, postnatal growth^52^, and cessation of reproduction^46^. All of these metrics influence the number of breeding attempts, number and growth of neonates and eventually dictate the annual reproduction output. Reproductive seasonality is known as the timing of reproduction across two or more consecutive years and synchrony of reproduction is considered as similarity in the timing of events within a population in a single year^53^. Seasonality and synchrony in reproduction can also be affected by temperature and levels of precipitation^52,53^. The degree of both reproductive synchrony and seasonality may be under influence of several factors such as timing of maximum resource availability, predator density, and advantages of intra-specific cooperation^53^.

To predict how bat community will react to future climatic changes, it is necessary to understand fully the effects of climatic variables on life-history traits, individual fitness and population viability, and how these differ between species. These information are also necessary to inform conservation planning. In the present study, we used 2 years (2015 and 2016) of mark-recapture data from a maternity colony of three sympatric bat populations (*Rhinolophus* *euryale*, *R*. *ferrumequinum*, and *Myotis* *emarginatus*) to investigate breeding phenology (the timing of biological events) at community level. We empirically tested hypotheses about the influence of climatic conditions (temperature and precipitation) on the patterns of phenological congregations, birth synchrony and seasonality and postnatal growth in a mixed nursing colony of three bat species. We predicted that spring weather conditions would have influence breeding phenology within our study populations. Specifically, we examined whether:

(1) There are associations between climate and phenologically measurable life-history traits in three bat species, (2) the timing of parturition and birth synchrony are affected by spring weather conditions. Cold and wet spring weather in 2016 was predicted to delay parturition and increase birth duration, whilst warm and dry spring conditions in 2015 were predicted to advance parturition dates and decrease duration of the parturition period, (3) among species, bats born in wet year would be smaller and grow more slowly than those born in dry year, (4) climate conditions affect timing of return to the cave nursing colony and drought and warm conditions are associated with returning earlier.

To evaluate responses of the bat community to an extreme climate event we use the following metrics: (1) changes in recruitment of the bat species to see if species assemble differently in the wet year. This is measured by time of first arrivals and also the peak of the neonate under postnatal study in both years. (2) Birth synchrony as measured by mean date and vector lengths of parturition dates in both dry and wet years. (3) Changes in the spontaneous growth rates as indicated by ratio of the changes in body mass and forearm length to change in unit of time.

## Materials and methods

### Study area and species

This study was conducted in Kerend cave (34° 15′ 36.7″ N, 46° 17′ 22.5″ E, 1620 m above sea level) from April to August in 2015 and 2016. The cave is located in mid-Zagros Range, Kermanshah province in western Iran. The climate in western edge of the Iranian Plateau is characterized by a pronounced seasonal variation, including a long freezing period in winter and mild summer. Most precipitation in the study area occurs during autumn, winter, and spring (October–May months). The cave has a single corridor and its entrance is approximately 2 m high and 5 m wide. A mixed colony of approximately 400 *Rhinolophus* *euryale*, 200 *R*. *ferrumequinum* and 250 M. *emarginatus* roosted in this cave as a nursing colony^54^. Average relative humidity and temperature in the cave during the study period were 60.14 ± 14% and 22.21 ± 0.98 °C, respectively.

The surrounding area of the cave is characterized by open woodland. However, the lowland below the cave is completely altered to various agricultural field but at higher elevation around the cave patches of natural vegetation (oak-pistachio) are still persisted. In these areas, oak woodland and other vegetation formations such as deciduous dwarf-scrublands, amygdales scrublands and cushion shrub land are present^54^.

The Mediterranean horseshoe bat (*Rhinolophus* *euryale*, Blasius, 1853) is a cave-dwelling medium-sized bat (body mass = 8–17.5 g; forearm length = 43–51 mm)^55^. The species is mainly distributed from northwestern Africa through most of southern Europe to the Middle East and the Caucasus^56^. The southern limits of its distribution range are in the Levant and Iran, while the northern limits extend to southern Slovakia and northern Hungary^57,58^. Its distribution in Iran is restricted to natural caves in western (throughout the Zagros Mts.) and northern (Elburz Mountains) parts of the country^56,59^. Status of this species classified as rare in Iran^60^ and near threatened (NT) on the global scale^61^. Previous studies on habitat and diet selection by the Mediterranean horseshoe bat have found that this species appears to be sensitive to urban sites and open areas, while it mainly selected broadleaved woodland and dense vegetation, with moths as the main prey^62–64^. However, Diptera (especially Tipulidae) and Coleoptera such as beetles could also play an important role in some habitats^59,65–67^. The diet of *R*. *euryale* in Iran has not yet been studied.

The Geoffroy’s bat (*Myotis* *emarginatus*, Geoffroy, 1806) is one of nine species of the mouse-eared bats, genus *Myotis* occurring in Iran^68^; categorized as medium-frequent bats in this country. Iranian records of *M*. *emarginatus* are available from both northern humid areas and southern arid lowlands of the country, with very different climatic conditions^59^. This species occurs in the north-west Africa and South Europe through Central and Mediterranean Europe to the Caucasus, southern Arabia, the eastern part of the Mediterranean, Iran, Afghanistan and west Turkestan^57,59,69^. Both molecular and morphologic studies have shown that the species feed on Coleoptera, Homoptera, Araneae, Lepidoptera, Brachycera Diptera, and Lepidoptera larvae^59,70^. However, spiders are the primary food source for this bat species in all the studied locations. Global conservation status of *M*. *emarginatus* classified as least concern (LC) on the IUCN^68,71^. In the conservation evaluation system in Iran classified as rare^60^.

The greater horseshoe bat (*R*. *ferrumequinum*, Schreber, 1774) is a relatively large insectivorous bat and is widely distributed in northern Africa and southern Europe to south-west Asia, the Caucasus, the Himalayas to south eastern China, Korea and Japan^72–74^. Species is distributed throughout Iran (99 localities), with the exceptions of the central deserts, including Dasht-e Kavir and Dasht-e Lut^59,75^. The study on reproduction synchrony and postnatal growth in *R*. *ferrumequinum* in two successive dry (2015) and wet year (2016) in Kerend cave has been conducted with current study, however, results of this study has been published in advance^40^.

### Sampling and climate data

To compare birth synchrony and seasonality in dry and wet years, upon the appearance of new borne pups, birth date for each species was recorded. We considered pups with an umbilicus still attached had been born in the previous 24 h^76^. For all pups without umbilical cord (unknown-age neonates), birth date was estimated using two age-predictive equations for body mass and forearm length obtained from postnatal growth curves. A ninety-five percent confidence intervals and prediction limits were plotted for the regression equations of body mass and forearm length to indicate whether these two lines can reflect the degree of variation in dry and wet years. In 2015 and 2016, we captured 28 and 24 one-day-old *M*. *emarginatus* pups for the postnatal study. Additionally, we captured 12 and 18 one-day-old *R*. *euryale* pups in 2015 and 2016 for the same purpose.

Using body mass and forearm length growth curves (1–20 days), we estimated the age of *M*. *emarginatus* pups that born late. The following regression equations were derived from the best-fitting line to the growth data on body mass and forearm length:

2015: Age (days) = 3.75 (body mass) − 9.38; Age (days) = 0.72 (Forearm length) − 12.58,

2016: Age (days) = 4.29 (body mass) − 10.69; Age (days) = 0.78 (Forearm length) − 10.98.

Age predictive equations that derived from body mass and forearm lengths of known-age *R*. *euryale* individuals (1–28 days) were as follows:

2015: Age (days) = 4.50 (body mass) − 20.19; Age (days) = 0.95 (Forearm length) − 22.56.

2016: Age (days) = 4.32 (body mass) − 16.19; Age (days) = 0.83 (Forearm length) − 15.58.

To measure growth rates, newborne pups were hand-captured immediately after evening emergence of lactating female. They were placed into cloth bags individually and their original location noted. Following sex determination (by inspecting external genital), pups were banded on their forearms with an individually numbered aluminium alloy band (0.05 g, 2.9 mm; Porzana Ltd, http://www.porzana.co.uk). Bats were weighed to the nearest 0.01 g with an electronic balance (Electronic Analytical Balance Scale SF-400C, Pakistan). Length of the forearm and total epiphyseal gap were measured to the nearest 0.01 mm with a digital calliper 200 ± 0.01 mm (LG Guanglu, HB-102-111, China). To measure length of the total epiphyseal gap, a strong torch was used to trans-illuminate the right wing to allow accurate measurement of the gap. In 2015, to avoid injury or mortality to delicate newborn *R*. *euryale*, we started our postnatal measurements and wing tracing when neonates have no umbilical cord, while for *M*. *emarginatus*, postnatal measurements started from day 1. Flightless young bats were then returned to their original location immediately after measurements. In order to maintain bat body odour, we used individual surgical gloves when handling the neonates. We did not observe any injury or adverse reaction to tagging, weighting, and wing tracing of pups.

Wing area (mm^2^) was measured by placing the young bat on its ventral side over on a 20 × 30 cm latticed paper having 1 × 1 mm grid, extending the right wing with the leading-edge perpendicular to the body axis and tracing the outline. The traced area was carefully scanned and converted to pictures in JPG format, which were used to calculate wing area and wingspan using Digimizer software, version 4.1.1.0 to the nearest 0.01 mm^2^ and 0.01 mm, respectively. Wingspan (mm) was calculated as two times the distance from the body axis to the wing tip. Wing loading (N m^−2^) was calculated by multiplying individual body mass by 9.8 (acceleration due to gravity) and dividing the result by the wing area. Aspect ratio was calculated as wingspan squared and divided by wing area.

Data on monthly weather variables per year were gathered from the closest synoptic weather station at Islamabad (c. 10 km away). We used monthly mean temperature (degrees Celsius) and total precipitation data (millimetres for January through August for each year). Also, 28-year information since establishment of this station was used to represent regional climate by a climograph integrating 28-year data on mean monthly precipitation and temperature. In addition, average monthly temperatures and relative humidity of the cave were recorded for both years from April through August, using a digital thermo-humidity meter (HTC-2 LCD digital temperature humidity meter, China).

### Instantaneous growth rate

In dry and wet years, instantaneous growth rate (the daily increment as a proportion of total size: $\frac{dX}{dt}$) of body mass, forearm length, wing area and wingspan were measured for 47 and 70 days in *M*. *emarginatus* and *R*. *euryale*, respectively. To measure the instantaneous growth rate, we used the formula proposed and validated by Krebs^77^ and Eghbali and Sharifi^40^ as follows:

1 $$\frac{dX}{dt}=\frac{rX(K-X)}{K}$$

where X is the mean growth variable, t is the time (days), r is the early exponential growth rate for the growth variable during pre-flight period, and K is the upper asymptote or maximal value of the variable X. The early exponential growth rate (r) was determined as the slope of a trendline fitted to the variable data in a graph in which the y-coordinate was ln [(K − X)/K], and the x coordinate was time^77^.

### Statistical analysis

Results were presented as mean ± SD (standard deviation). We accepted comparisons as statically significant at α = 0.05. Data on local mean monthly temperatures and precipitation were compared between years using t-test and one-way ANOVA for normally distributed variables. Additionally, we tested for differences in average monthly temperatures and relative humidity of the cave between the 2 years using t-test. Mann–Whitney U-test used for variables that were not normally distributed. Normality was assessed with the Shapiro–Wilk method. We used SAS version 9.2 (SAS Institute Inc., Cary, North Carolina) and SPSS version 20.0 (IBM SPSS, IBM Corp, Armonk, New York) for statistical analysis.

For differences between years in variables at birth and in growth rates, we used the linear phase of growth curves (pre-flight stage) because the complete empirical growth curves for both years are curvilinear. For each growth variable, we compared the years using multiple regression and generalized estimating equation (GEE) models that include year as a grouping factor, age as a covariate, with an interaction term (age by year). For each model, we used the partial F-statistic for the interaction term to perform a test for parallelism to compare the slopes describing growth rates (variable vs. age) for each year and the partial F-statistic for the group term to perform a test for equal intercepts to compare the estimated size at day 1 (y-intercept) of each growth variable for each year. For estimating age of young at different growth stages and recording birth date of juveniles, we applied the linear regression of the growth parameters (forearm length and body mass) against age as dependent variable during pre-flight period. Furthermore, we tested for differences birth duration between years and among species using ANOVA with Tukey’s test for pairwise comparisons.

Birth synchrony and seasonality of each species were analysed using circular statistics^40,53^ in ORIANA 4.2 and R 3.5.1 software with significance at P < 0.05. Here, synchrony was defined as the clustering of births within a year, and seasonality was considered as the tendency for clusters of births to fall at approximately the same point in dry and wet years. Circular statistics considers year as a circle, with each of the 365 days of the year as a point on the circle. Therefore, any birth can be quantified as vector (mean) representing the number of points (days) reporting the event. The mean and variance around the mean length of mean vector, mean angles and the angular variance are computed by Batschelet^78^, Heideman and Utzurrum^53^ and Eghbali and Sharifi^40^ as follows:

2 $$\overline{x }=\frac{1}{n}\sum_{i=1}^{ n}\mathrm{cos}({\varnothing }_{i})$$

3 $$\overline{y }=\frac{1}{n}\sum_{i=1}^{n}\mathrm{sin}({\varnothing }_{i})$$

where ${\varnothing }_{i}$ (angle) were the dates in radiations of births from a data set. The length of the mean vector was calculated from following equation:

4 $$r=\sqrt{{\overline{x} }^{2}+{\overline{y} }^{2}}$$

The mean angles ($\overline{\varnothing  })$ and the angular variance (*S*^2^) were calculated using following equations:

5 $$\overline{\varnothing  }=\left\{\begin{array}{c}\mathrm{arctan}\left(\frac{\overline{y} }{\overline{x} }\right);\overline{x }>0 \\ \pi +\mathrm{arctan}\left(\frac{\overline{y} }{\overline{x} }\right);\overline{x }<0\end{array}\right.$$

6 $$\mathrm{S}^{2}= 2 (1 -\mathrm{ r})$$

### Ethical approval

Bats were captured and handled in the field in accordance with guidelines approved by the Razi University Animal Care Committee (Ethic certificate No. 396-2-003). All experiments were performed in accordance with relevant guidelines and regulations. Our reporting of research involving animals follows the recommendations of the ARRIVE guidelines.

## Result

### Local weather conditions

**Figure 1:**
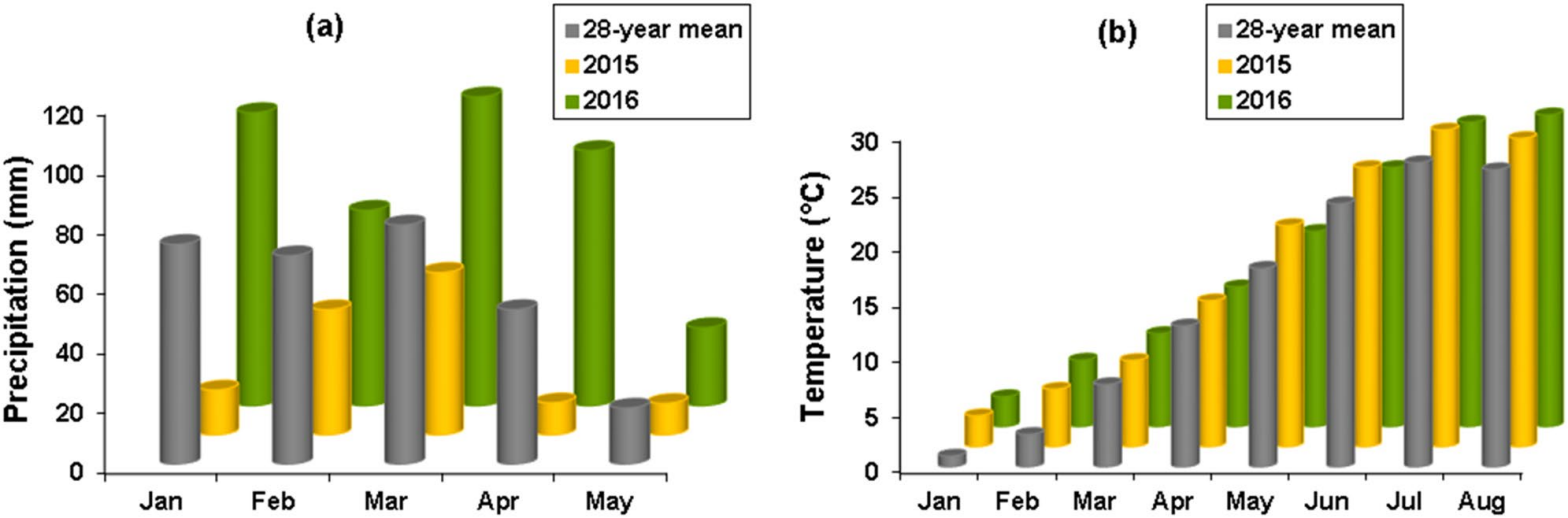
A chart bar demonstrating regional climate based on available data collected in Islamabad synoptic station at about ten kilometres distance to the Kerend cave. Total monthly precipitation for January–May in 2015, 2016 and 28-year average (**a**; redrawn form^40^). Monthly average temperatures for January–August in 2015, 2016 and in 28-year average (**b**; reproduced data points from^40^). Grey columns indicate regional monthly averages from 1987 to 2014 for precipitation (**a**) and temperature (**b**).

Local climate conditions in the study area during January to August (2015–2016) are shown in Fig. 1. Total precipitation data showed inter-year significant variation (Fig. 1a). Total monthly rainfall for each month from January to May in 2016 was also significantly more than similar data in 2015 (P < 0.05). In addition, for all months except February, average monthly precipitation in 2016 was higher than average data available for 28 years from 1987 to 2014. The variation in mean monthly temperatures were lower than precipitation among the 2 years. The overall mean monthly temperature in January through August were higher than normal (28-year mean for local temperatures) in dry year. Likewise, as shown in Fig. 1b, during the prenatal growing month (May) and the two postnatal growing periods (June and July), the monthly temperatures were significantly different among dry and wet years (May: t = 3.58, P = 0.001; June: t = 3.25, P = 0.002; July: t = 2.87, P = 0.006), but non-significant differences were found between dry and wet years for April and August (April: t = 0.99, P = 0.32; August: Mann–Whitney test: z = − 0.79, P = 0.42; Fig. 1b).

**Figure 2:**
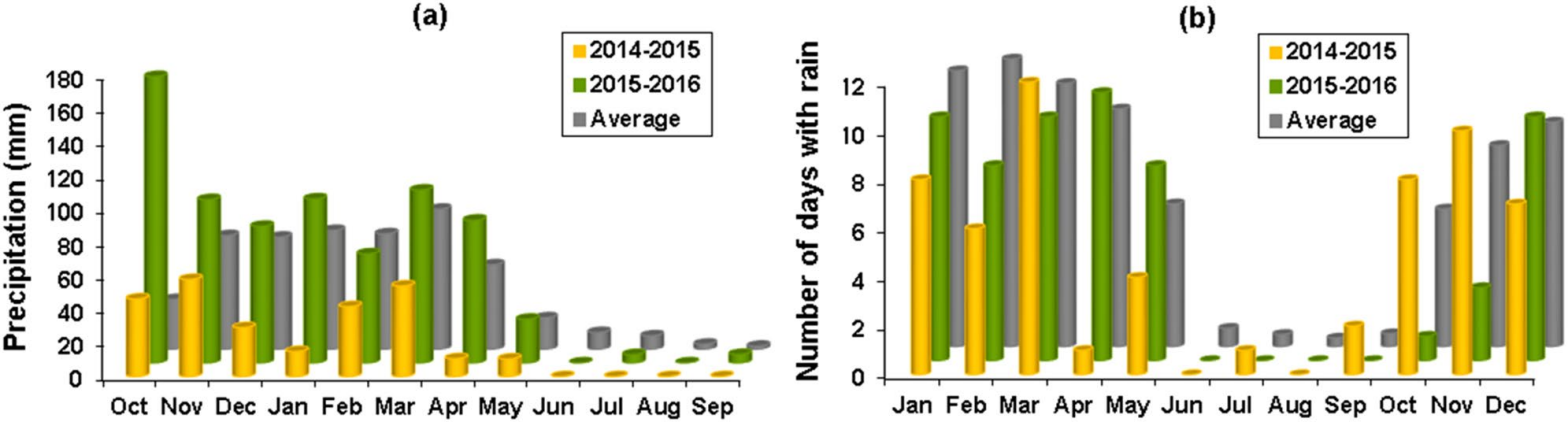
Monthly changes in precipitation for 2014–2015, 2015–2016 and average for 28 years (1987–2014) covering October–September (**a**) and the number of days with measurable rain for 2015, 2016 and average for 28 years (**b**) (redrawn form^40^).

Available information on the climatic conditions in the study area are limited to data collected in Islamabad synoptic station the closest station to the Kerend cave during 1987–2016. These data have been used to provide basic information for climatic conditions in the same study area by Eghbali and Sharifi^40^. Based on this explanation the climatic conditions in the study area is described as a typical cold Mediterranean with a short freezing winters and mild summers. In this area a long and dry season extends from June to September with no precipitation and most rainfalls fall during October to May (Fig. 2a). Average annual precipitation during 28 years (1987–2014) is 462 mm. For the same time average annual temperature is 14.18 ± 0.92 °C, ranging from 0.97 ± 2.65 °C in January (the coldest month) to 27.45 ± 1.37 °C in July. In 2015 these values were 15.15 °C and 277.3 mm and in the wet year were 14.64 °C and 729.6 mm. Average monthly precipitation in 2014–2015, 2015–2016 and average long term (28 years) are shown in Fig. 2a. An analysis of rainfall data for the pregnancy period (April and early May) revealed that monthly precipitation recorded (Fig. 2a) were significantly higher by seven times in 2016 (142.20 mm) than in 2015 (19.80 mm). The local temperatures also differed significantly between the two study years. Average number of days with measurable rains for 28 years, 2015 and 2016 are shown in Fig. 2b.

### Cave climate

Relative humidity (%) and temperatures (°C) in Kerend cave were compared from April to August between years. The mean monthly relative humidity (%) in the cave was significantly different among dry and wet years for April–August (all P > 0.05). Furthermore, the thermal conditions of the cave also differed remarkably between years for May and June (May: t = 2.39, df = 10, P = 0.03; June: t = 2.66, df = 8, P = 0.02, see^40^). The mean monthly temperature was not significantly different among 2 years for (April: t = 1.00, df = 4, P = 0.37; July: t = 0.77, df = 6, P = 0.46 and August: t = 2.00, df = 4, P = 0.11, see^40^).

### Phenological recruitment

In both years, *R*. *euryale*, *R*. *ferrumequinum* and *M*. *emarginatus* assembled in the Kerend cave for reproduction. The timing of arrival, date of first birth record, peak of postnatal growth, upper asymptote for growth curve, time of first foraging flight and finally time of departure from the nursing roost for the three bats in 2015 and 2016 are shown in Table 1. All neonates belonging to the three species were congregated in a single cluster. Reassembly of bats in 2016 included the same species but timing of arrival, peak of postnatal growth, first foraging flight for neonates and departure from the nursing roost were comparable (Table 1). As indicated in Table 1, in 2015, first pregnant *R*. *euryale* arrived into the cave on 10th of April and first parturition was recorded on 24th of May. In 2016, this species entered the cave about 5 days later (15th April) and gave birth to the first neonate on 20th of May. In 2016, *M*. *emarginatus* entered the nursing cave on 20th April and gave birth to the first neonate on 3th of June. Despite giving birth earlier, a reduction in postnatal growth period as indicated by leveling of the non-linear growth curve began in *R*. *euryale* on 28 days after birth in 2016, 4 days longer than 2015. In this species, on average first foraging flights in 2016 began 5 days later than in 2015. No difference was found in the non-linear growth curves for *M*. *emarginatus* in 2015 and 2016. This species, also, did not show any significant differences in the level of growth asymptote and times of the first foraging flights in 2015 and 2016 (Table 1).

**Table 1:** Timing of major phenological events in dry (2015) and wet (2016) years for *Rhinolophus* *euryale*, *Rhinolophus* *ferrumequinum* (see^40^) and *Myotis* *emarginatus* (see^79^).

Phenological events	*Rhinolophus* *euryale*		*Myotis* *emarginatus*		*Rhinolophus* *ferrumequinum*	
	2015	2016	2015	2016	2015	2016
First arrival seen	10 Apr	15 Apr	15 Apr	20 Apr	17 Apr	22 Apr
First birth record	24 May	20 May	1 Jun	3 Jun	20 May	26 May
Peak of postnatal growth	Until 24 days	Until 28 days	Until 20 days	Until 20 days	Until 24 days	Until 30 days
Asymptotic size (g)	10.28 ± 0.19	10.38 ± 0.22	7.43 ± 0.16	7.15 ± 0.28	17.74 ± 0.67	16.75 ± 0.49
First foraging flight (days)	40–45	45–50	35–40	35–40	40–45	45–50
Growth constant	0.08	0.10	0.22	0.19	0.20	0.19

### Birth synchrony and seasonality

**Figure 3:**
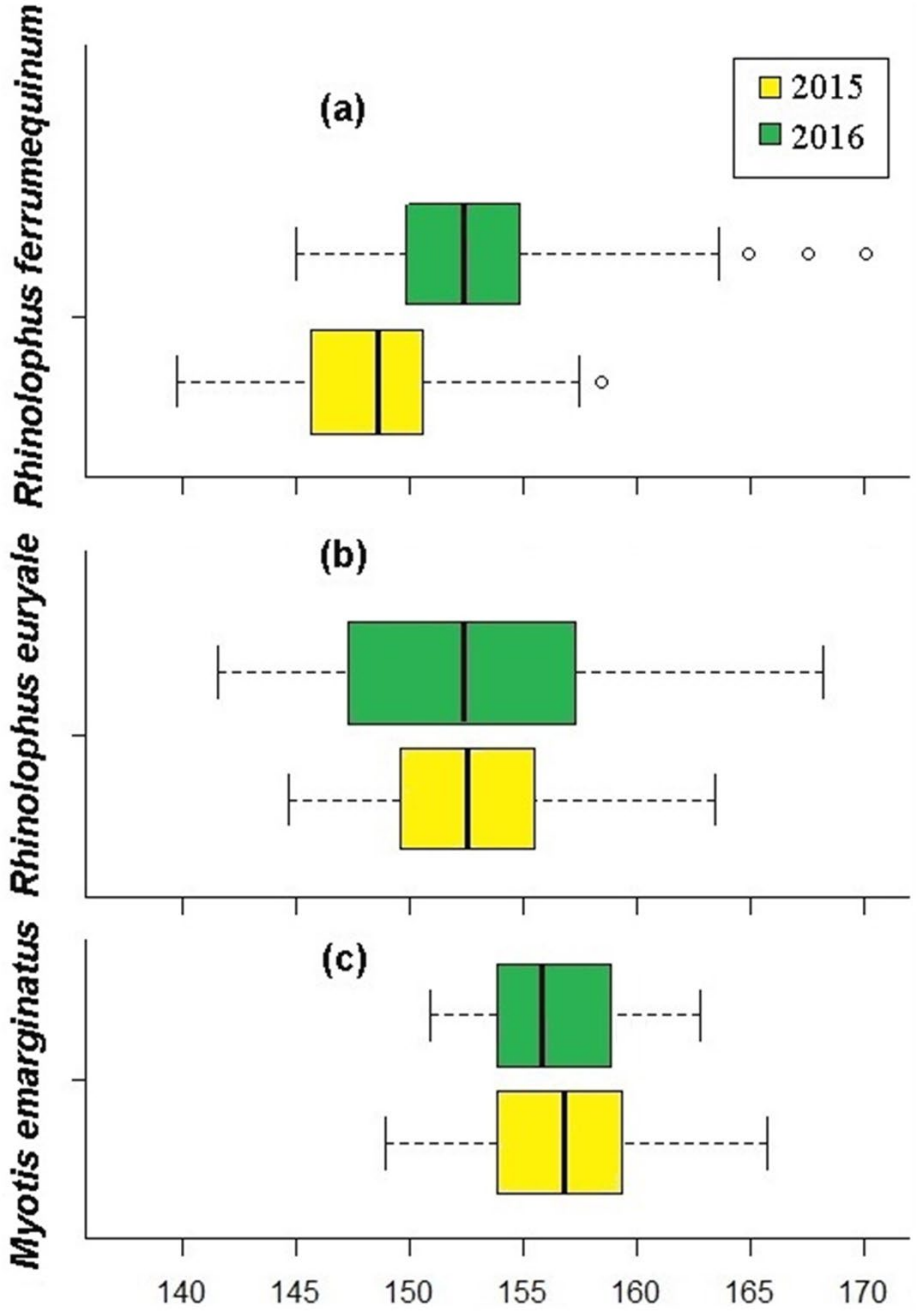
Box plot showing birth timing of *Rhinolophus* *ferrumequinum* (**a** redrawn form^40^) *Rhinolophus* *euryale* (**b**), and *Myotis* *emarginatus* (**c**) in 2015 and 2016 (20 May = Day 140). The line across the box indicates the median and the box shows the interquartile range. The ends of the whiskers represent maximum and minimum values, excluding outliers and extremes. Outliers between 1.5 box lengths and 3 box lengths from the end of the box are indicated by a circle.

In 2015 and 2016, 189 and 193 births occurred in *R*. *euryale*. These values for *M*. *emarginatus* were 139 and 138, respectively. For *R*. *ferrumequinum*, in 2015, a total of 113 births were recorded during 20 days after first parturition on 20 May (Fig. 3). In 2016, a total of 96 greater horseshoe bat gave birth during 28 days after first parturition on 26 May. The estimated births were significantly seasonal (r = 0.99; Table 2) for all species and were clumped in one well-defined cluster per year (late May–early June), but differences were found in the degree of birth synchrony and the timing of births between the bat community in 2015 (ANOVA: F = 308.751; P < 0.001), as well as in 2016 (ANOVA: F = 31.409; P < 0.001). Pups in *R*. *ferrumequinum* were born significantly earlier in the cluster of 2015 than pups in 2016 (Tukey post hoc test: P < 0.05; Fig. 3a) by an average of about 7 days. In 2015 and 2016, *R*. *euryale* births occurred earlier compared to *M*. *emarginatus* (Tukey post hoc test: P < 0.001; Fig. 3). In addition, the duration of the delivery period varied between 22 days in *R*. *euryale* and 18 days in *M*. *emarginatus* in 2015. A significant difference was also found between *R*. *euryale* and *M*. *emarginatus* (28 days vs. 13 days), with a longer birthing period in 2016 for the Mediterranean horseshoe bat (Mann–Whitney test: z = 5.0; P < 0.001; Fig. 3b,c).

**Table 2:** Output of circular statistics used in analyses of synchrony and seasonality of parturition in the studied nursing colony of *Rhinolophus* *euryale*, *Rhinolophus* *ferrumequinum* (see^40^), and *Myotis* *emarginatus* in two successive dry (2015) and wet year (2016).

Species	Year	x.Min (°)	x.Max (°)	x.Median (°)	x.Mean (°)	P*	$r$	$\overline{y }$	$\overline{x }$	${S}^{2}$
*Rhinolophus* *euryale*	2015	146.95	165.69	154.84	154.80	**0.001**	0.997	0.422	− 0.903	0.004
	2016	144.00	170.63	154.84	155.15		0.994	0.415	− 0.903	0.01
*Myotis* *emarginatus*	2015	148.93	165.69	156.82	158.69	0.079	0.998	0.394	− 0.916	0.003
	2016	150.90	162.73	155.83	159.72		0.998	0.395	− 0.917	0.002
*Rhinolophus* *ferrumequinum*	2015	137.09	155.83	145.97	145.73	**0.028**	0.998	0.561	− 0.824	0.003
	2016	143.01	169.64	150.41	151.18		0.996	0.480	− 0.873	0.006

Significant values are in [bold].

High values of “r” reflect strong seasonality of births; low values of “S^2^” reflect lower angular variance indicating greater clustering in births; *P value from ANOVA and used for comparing the duration of births in dry and wet years for each species.

**Figure 4:**
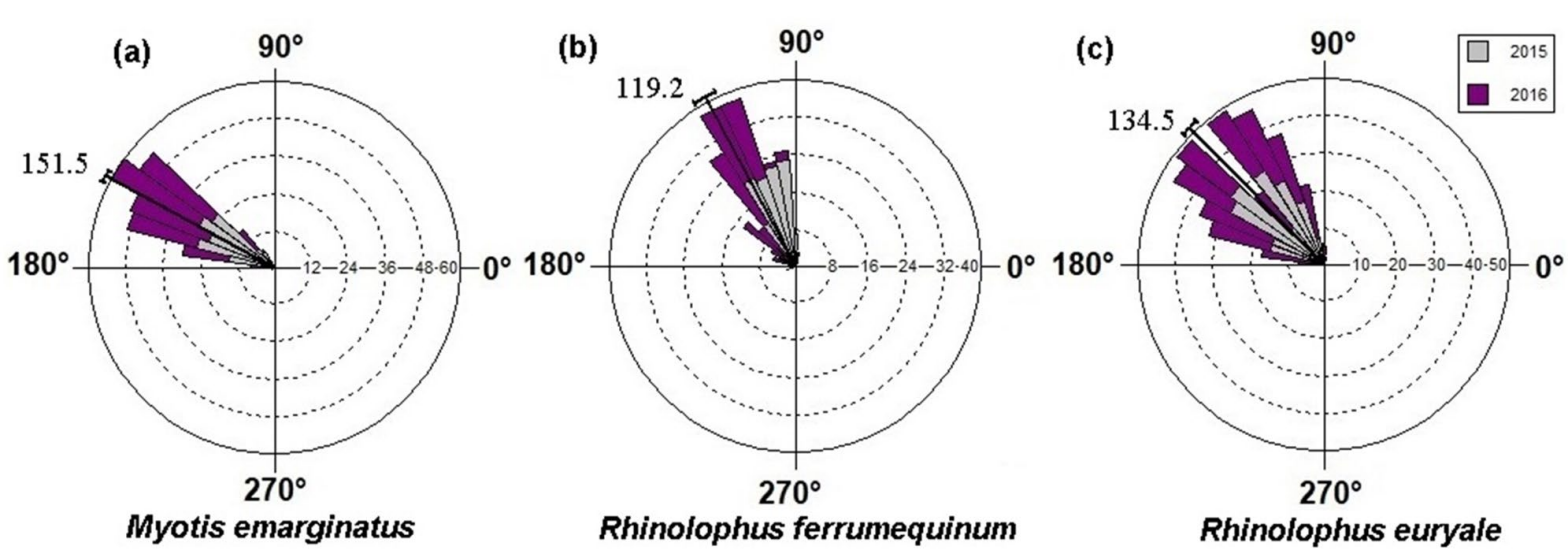
Circular histograms of the frequencies of births of three bat species, *Myotis* *emarginatus* (**a**), *Rhinolophus* *ferrumequinum* (**b** redrawn form^40^) and *Rhinolophus* *euryale* (**c**) in two successive dry (2015) and extreme wet (2016) events. The vector line in the circle indicates the grand mean vector and the sector outside the circle indicates the 95% confidence interval. Here, the circular scale defined as May, June and July months, where 20 May ~ 77°.

Although estimated dates of birth in *R*. *euryale* were both highly synchronous and seasonal, but the pattern of delivery timing recorded for the dry year yielded different results compared to those obtained from the wet year records (Fig. 4; Table 2). In 2015, 81.0% of the 189 recorded *R*. *euryale* births occurred in 14-day period between 26 May and 8 June, as compared to 56% of the 193 recorded births occurred in the same period between 24 May and 5 June. Number of pups born in 2015 were 1–17 (mean = 8.6) per day. In 2016, the number of births ranged from 2–13 (mean = 6.9) per day. Based on the angular variance (S^2^), *R*. *euryale* had significant synchrony of births in 2015 (S^2^ = 0.004) than 2016 (S^2^ = 0.01). Despite the strong seasonality of births in dry and wet years (r = 0.99), the duration of parturition differed significantly between the years (2015: x Min = 146.95°, x Max = 165.69°; 2016: x Min = 144.00°, x Max = 170.63°; P < 0.001; Table 2). The Mediterranean horseshoe bat gave birth to their first pup on 24 May in 2015 and 20 May in 2016 and the last recorded delivery on 18 June and 15 June, respectively. Thus, pups were born earlier in 2016 but experienced an extended period of delivery than those born in 2015. However, despite this shift between years, the pattern of differences in mean birth dates remained the same among the dry and wet years, so the mean angles of birth dates based on circular statistics in the dry and wet years are 154.80^◦^ and 155.15^◦^, respectively, which is equal to the mean birth date on 4 June (Table 2; Fig. 4).

The Geoffroy’s bat *M*. *emarginatus* compared with *R*. *euryale* and *R*. *ferrumequinum* gives birth late in both dry and wet years (Fig. 4; Table 2), but had significant synchrony of births within its population in the cave in each year, so that all births occurred in the 18-day period in 2015 and 13-day period in 2016 (2015: x Min = 148.63°, x Max = 165.69°; 2016: x Min = 150.90°, x Max = 162.73°; Table 2). Therefore, birth synchrony did not decline over 2 subsequent years. In addition, this species has a highly seasonal pattern of reproduction. The mean angles of birth dates in the dry and wet years are 158.69° and 159.72°, respectively, which is equal to the mean birth date on 8 June (Fig. 4). This species gave birth to their first pup on 1 June in 2015 and 3 June in 2016 and by 10 days had 66% and 87% of neonates were born in dry and wet years, respectively. The average number of births per day ranged from 1 to 15 (mean = 7.72) in 2015 and 1 to 17 (mean = 10.61) in 2016. However, unlike *R*. *euryale*, our data for the Geoffroy’s bat indicated that the duration of the birthing period not varied between the dry and wet years (P = 0.079; Table 2).

In *R*. *ferrumequinum*, despite the strong seasonality of births each year (r = 0.99; Table 2), mean parturition date differed significantly between dry and wet years. Pups were born significantly later in 2016 (mean = 4 June) than in 2015 (mean = 28 May; Tukey post hoc test: P < 0.05). Moreover, the duration of parturition period varied between 20 days in 2015 and 28 days in 2016 (2015: x Min = 137.09°, x Max = 155.83°; 2016: x Min = 143.01°, x Max = 169.64°; Table 2). A significant difference was found between years, with a longer birthing period in 2016 (P < 0.05). However, based on the results obtained from the circular statistics, difference in angular variance of clustering of births was lower in the original data than in 95% of randomized iterations for the 2 years (P < 0.05) and thus births in both years were significantly synchronous (Fig. 4). Lower angular variance of birth dates in data set of 2015 indicates greater clustering. On the other hand, this value suggests that the level of synchrony of parturition was higher in the dry year (2015: S^2^ = 0.003; 2016: S^2^ = 0.006). Latest births of a few individuals in 2016 occurred on the last 10 days of June (Fig. 3a). The average number of births per day ranged from 1 to 13 (mean = 5.65) in 2015 and 1 to 11 (mean = 3.42) in 2016.

### Size at birth and postnatal growth

In the three species 1-day-old pups were altricial and thus they were naked, pink in colour, with closed eyes and folded pinnae. The pups positioned themselves firmly on the ventral side of their mothers. For the Mediterranean horseshoe bat (*R*. *euryale*), in 2015, the forearm length of pups at 4-day-old ranged from 24.89 to 28.43 mm ($\overline{x }$ = 26.16 ± 1.31 mm), body mass ranged from 4.06 to 5.65 g ($\overline{x }$ = 4.94 ± 0.49 g), wing area ranged from 5510.00 to 7323.00 mm^2^ ($\overline{x }$ = 6595.41 ± 560.50 mm^2^), wingspan ranged from 164.00 to 187.00 mm ($\overline{x }$ = 177.16 ± 8.14 mm), and dimension of wing loading varied from 6.70 to 10.72 N m^−2^ ($\overline{x }$ = 8.20 ± 1.24 N m^−2^). In 2016, the forearm length of pups at 1-day-old ranged from 14.45 to 21.55 mm ($\overline{x }$ = 19.40 ± 1.26 mm) and reached to 23.51 ± 1.78 mm at 4 days, and body mass ranged from 3.55 to 4.10 g ($\overline{x }$ = 3.85 ± 0.15 g) and reached to 4.60 ± 0.36 g at 4 days. The wingspan of pups at 1-day-old ranged from 116.67 to 143.53 mm ($\overline{x }$ = 133.77 ± 7.97 mm) and reached to 150.55 ± 7.20 mm at 4 days, and dimension of wing area varied from 2685.17 to 3437.02 mm^2^ ($\overline{x }$ = 3106.07 ± 244 mm^2^) and reached to 3977.95 ± 414.16 mm^2^ at 4 days. The mean wing loading of 1-day-old pups varied from 8.62 to 11.93 N m^−2^ and reached to 10.18 ± 0.71 N m^−2^ at 4 days.

The mean body mass, forearm length and other wing characteristics, except for wing loading and length of epiphyseal gap, increased in a linear fashion during the early stages of postnatal growth period in 2015 and 2016 and thereafter they became non-linear. Wing loading decreased linearly until 28 days, which coincided with the attainment of clumsy flight and subsequently followed by a linear increase until end of postnatal period. However, up to eight recapture occasions, when the pups age at 28 days, in 2015, the pups attained the forearm length of 48.39 ± 0.65 mm, body mass of 9.36 ± 0.40 g, wing area of 14,446.44 ± 788 mm^2^ and wingspan of 298. 00 ± 9.79 mm which were 93%, 82%, 91% and 92% of postpartum females, respectively. These values for *R*. *euryale* in 2016 were 47.08 ± 1.33 mm, 8.95 ± 0.45 g, 12,540.95 ± 742 mm^2^ and 288.48 ± 10.62 mm respectively, which were 90%, 79%, 80% and 87% of postpartum females. Thus, the growth of forearm was fast compared to other growth variables in both years. Pups obtained 89% and 100% of mean sizes of wing loading of postpartum females at the age of 28 days in 2015 and 2016, respectively.

In 2015, mean length of forearm of 1-day-old Geoffroy’s bats measured after birth was 17.63 mm (SD = 1.63, n = 28) and body mass 2.58 g (SD = 0.31, n = 28). These values for wing area and wingspan 2417.88 mm^2^ (SD = 408, n = 28) and 112.82 mm (SD = 11.87, n = 28), respectively. In 2016, the average length of forearm was 17.37 mm (SD = 1.60, n = 24) and body mass 2.57 g (SD = 0.33, n = 24). The 1-day-old pups had an average wing area of 2319.69 mm^2^ (SD = 437, n = 24) and wingspan 112.12 mm (SD = 11.98, n = 24). During initial stages of postnatal growth (1–20 days) for both dry and wet years, body mass, forearm length, wingspan and wing area increased linearly. In 2015, when the pups were 3 weeks old, they attained the forearm length of 39.97 ± 1.25 mm, body mass of 6.81 ± 0.46 g, wing area of 11,473.61 ± 1024 mm^2^ and wingspan of 248.95 ± 15.72 mm which were 95%, 78%, 95% and 95% of postpartum females, respectively. These values for *M*. *emarginatus* in 2016 were, 38.12 ± 0.77 mm, 6.26 ± 0.32 g, 10,527.20 ± 740 mm^2^ and 236.70 ± 7.70 mm respectively, which were 91%, 72%, 79% and 90% of postpartum females. Also, there was no significant difference between 20-day-old pups in 2015 (5.96 ± 0.50 N m^−2^) and 2016 (5.99 ± 0.68 N m^−2^) in wing loading (Independent Samples Test, t = − 0.11, df = 28, P = 0.90).

### GEE analysis

**Figure 5:**
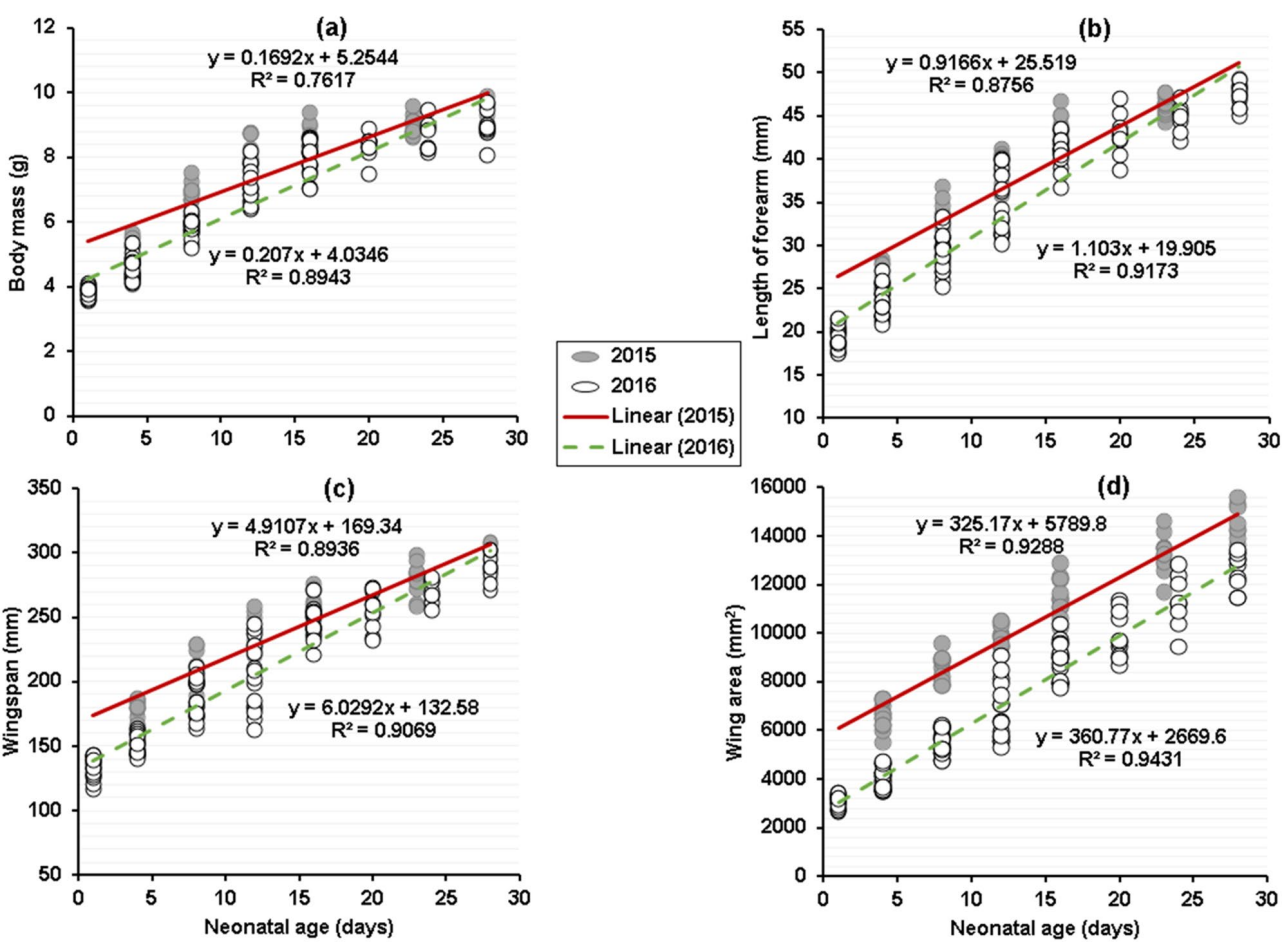
A comparison of age versus size in *Rhinolophus* *euryale* in 2 years, based on the early linear portion of postnatal growth (days 1–28). 2015 is represented by closed circles and a solid line and 2016 is represented by open circles and a dashed line. (**a**) Age versus body mass (GEE slope, P < 0.0001), (**b**) length of forearm (GEE slope, P < 0.0001), (**c**) wingspan (GEE slope, P < 0.0001), and (**d**) wing area (GEE slope, P = 0.85).

In the Mediterranean horseshoe bat, contrary to our initial prediction bats born in 2015 grew significantly slower than those born in 2016 (Table 3; Fig. 5). Based on multiple regression and generalized estimating-equation (GEE) analyses, in *R*. *euryale*, for body mass, forearm length, wingspan and wing loading tests of parallelism (interaction term) and tests for equal intercepts (group term) were significant (P < 0.001; Table 3). Thus, for each growth variable, the growth rate lines start with different intercepts and the lines diverge as pups aged (Fig. 5). For wing area, tests for equal intercepts were significantly different (P < 0.001), while tests of parallelism (interaction term or growth rate) were not (P > 0.05; Table 3). Thus, the growth rate lines start with different intercepts, but are parallel (Fig. 5d).

**Table 3:** Results of multiple regression and generalized estimating equation (GEE) analysis of growth parameters on age (as a covariate), group (sampling method), and an interaction (age × group) in *Rhinolophus* *euryale*, *Myotis* *emarginatus* and *Rhinolophus* *ferrumequinum* (reproduced data from^40^).

Growth parameter	Source of variation	*Rhinolophus* *euryale*				*Myotis* *emarginatus*				*Rhinolophus* *ferrumequinum*			
		Multiple regression		GEE		Multiple regression		GEE		Multiple regression		GEE	
		*df*^a^	*P*	*df*^a^	*P*	*df*^a^	*P*	*df*^a^	*P*	*df*^a^	*P*	*df*^a^	*P*
Body mass	Group	1	< 0.0001	1	< 0.0001	1	0.3214	1	0.1152	1	0.0570	1	0.0530
	Age	1	< 0.0001	1	< 0.0001	1	< 0.0001	1	< 0.0001	1	< 0.0001	1	< 0.0001
	Interaction (age by group)	1	0.0090	1	< 0.0001	1	0.0172	1	0.0104	1	< 0.0001	1	< 0.0001
	Residual	185		30^b^		248		52^b^		204		37^b^	
Length of forearm	Group	1	< 0.0001	1	< 0.0001	1	0.0061	1	0.0012	1	0.1087	1	0.1038
	Age	1	< 0.0001	1	< 0.0001	1	< 0.0001	1	< 0.0001	1	< 0.0001	1	< 0.0001
	Interaction (age by group)	1	0.0068	1	< 0.0001	1	0.4980	1	0.3628	1	< 0.0007	1	< 0.0005
	Residual	185		30^b^		248		52^b^		204		37^b^	
Length of epiphyseal gap	Group	1	0.0234	1	< 0.0001	1	0.1000	1	0.0978	1	0.2066	1	0.1999
	Age	1	< 0.0001	1	< 0.0001	1	< 0.0001	1	< 0.0001	1	< 0.0001	1	< 0.0001
	Interaction (age by group)	1	0.6155	1	0.4693	1	0.2642	1	0.1838	1	0.0382	1	< 0.0342
	Residual	143		50^b^		250		50^b^		178		33^b^	
Wingspan	Group	1	< 0.0001	1	< 0.0001	1	0.4110	1	0.4314	1	0.9028	1	0.9017
	Age	1	< 0.0001	1	< 0.0001	1	< 0.0001	1	< 0.0001	1	< 0.0001	1	< 0.0001
	Interaction (age by group)	1	0.0007	1	< 0.0001	1	0.0552	1	0.0243	1	< 0.0001	1	< 0.0001
	Residual	185		30^b^		248		52^b^		204		37^b^	
Wing area	Group	1	< 0.0001	1	< 0.0001	1	0.7354	1	0.6721	1	0.7583	1	0.7557
	Age	1	< 0.0001	1	< 0.0001	1	< 0.0001	1	< 0.0001	1	< 0.0001	1	< 0.0001
	Interaction (age by group)	1	0.8831	1	0.8507	1	< 0.0001	1	0.0001	1	< 0.0001	1	< 0.0001
	Residual	185		30^b^		248		52^b^		204		37^b^	
Wing loading	Group	1	< 0.0001	1	< 0.0001	1	0.8130	1	0.8780	1	0.4674	1	0.4622
	Age	1	< 0.0001	1	< 0.0001	1	< 0.0001	1	< 0.0001	1	< 0.0001	1	< 0.0001
	Interaction (age by group)	1	0.0002	1	< 0.0001	1	0.7977	1	0.7609	1	< 0.0281	1	< 0.0256
	Residual	185		30^b^		248		52^b^		204		37^b^	

^a^For residual sources of variation; ^b^numbers of clusters used in GEE analysis, not degrees of freedom (a cluster is a set of observations for 1 individual).

**Figure 6:**
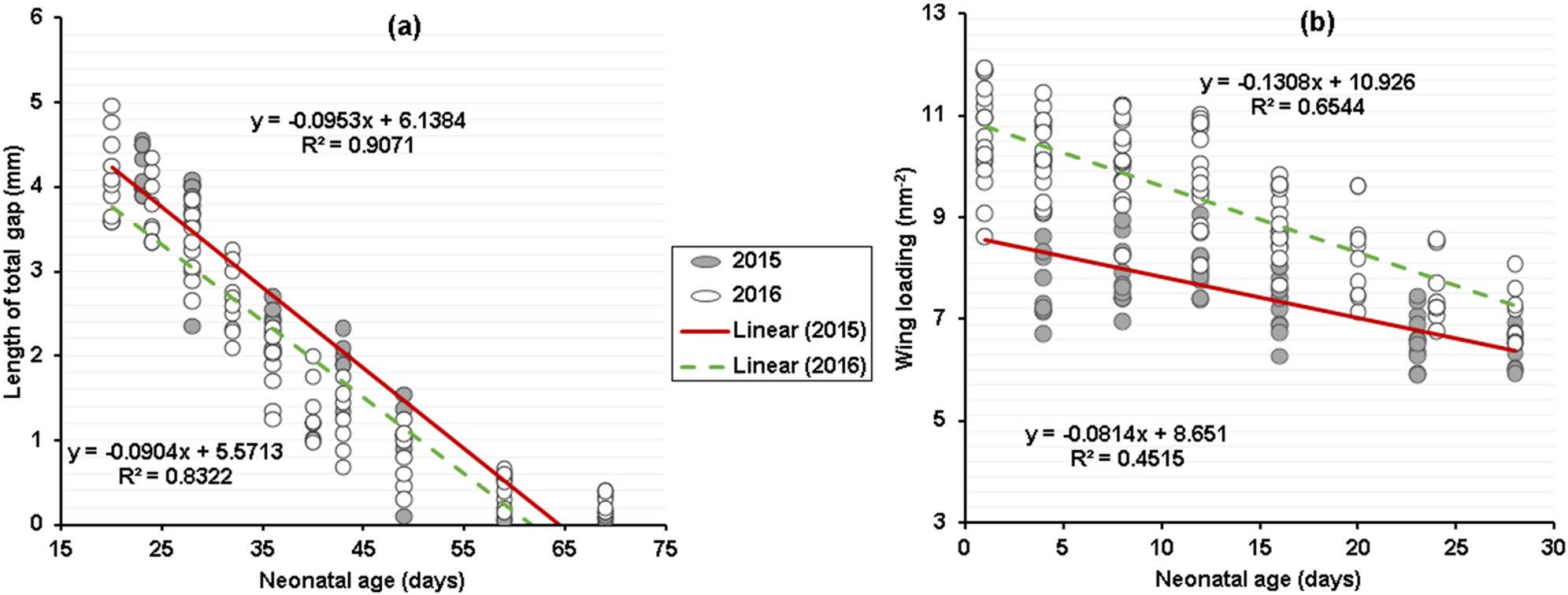
A comparison of age versus size in *Rhinolophus* *euryale* in 2 years, based on the latter linear phase of postnatal growth of the length of total gap (days 20–69) and the early linear portion of postnatal growth of wing loading (days 1–28). 2015 is represented by closed circles and a solid line and 2016 is represented by open circles and a dashed line.

In the later portion of the postnatal growth period (20–70 days), the length of the total epiphyseal gap decreased linearly (Fig. 6a). However, the initial sizes (y-intercepts) were significantly different (P < 0.001), while rates of growth (slope of variable on age) of this parameter did not differ between the 2 years (P > 0.05; Table 3). For wing loading, the rate of changes was significantly different between years (GEE slope, < 0.0001; Fig. 6b).

**Figure 7:**
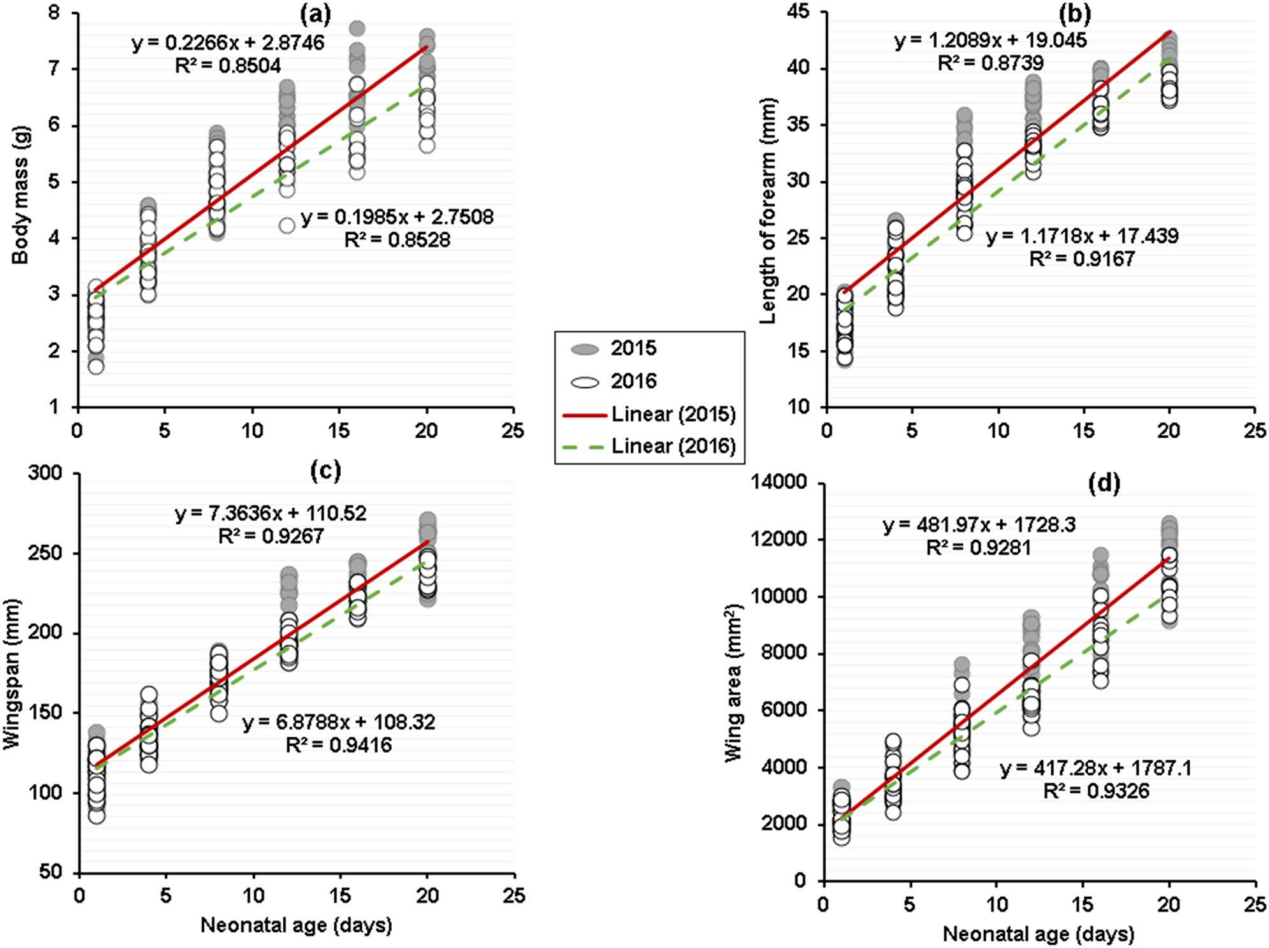
A comparison of age versus size in *Myotis* *emarginatus* in 2 years, based on the early linear portion of postnatal growth (days 1–20, see^79^). 2015 is represented by closed circles and a solid line and 2016 is represented by open circles and a dashed line. (**a**) Age versus body mass (GEE slope, P = 0.01), (**b**) length of forearm (GEE slope, P = 0.36), (**c**) wingspan (GEE slope, P = 0.02), and (**d**) wing area (GEE slope, P < 0.0001).

In the Geoffroy’s bat (*Myotis* *emarginatus*), agree to our initial prediction bats born in 2015 grew significantly faster than those born in 2016 (Table 3; Fig. 7). However, multiple regression and generalized estimating-equation (GEE) analyses gave similar results for each variable tested (Table 3). For body mass, wingspan and wing area tests for equal intercepts (group term or y-intercepts) did not differ between the 2 years (body mass, P = 0.11; wingspan, P = 0.43; wing area, P = 0.67; Table 3), while tests of parallelism (interaction term or slope of variable on age) were significant (P < 0.05). Thus, for each variable, the growth rate lines start with similar intercepts, but with the lines diverge as young aged (Fig. 7), with 2015 cohort yielding higher apparent growth rates for these growth variables than 2016 cohort. Similarly, forearm length increased linearly until 20 days of age in 2015 and 2016 (Fig. 7). However, for this variable, based on multiple regression and GEE analyses, tests for equal intercepts were significant (P = 0.001; Table 3), while tests of parallelism (interaction term) were not (P = 0.36).

**Figure 8:**
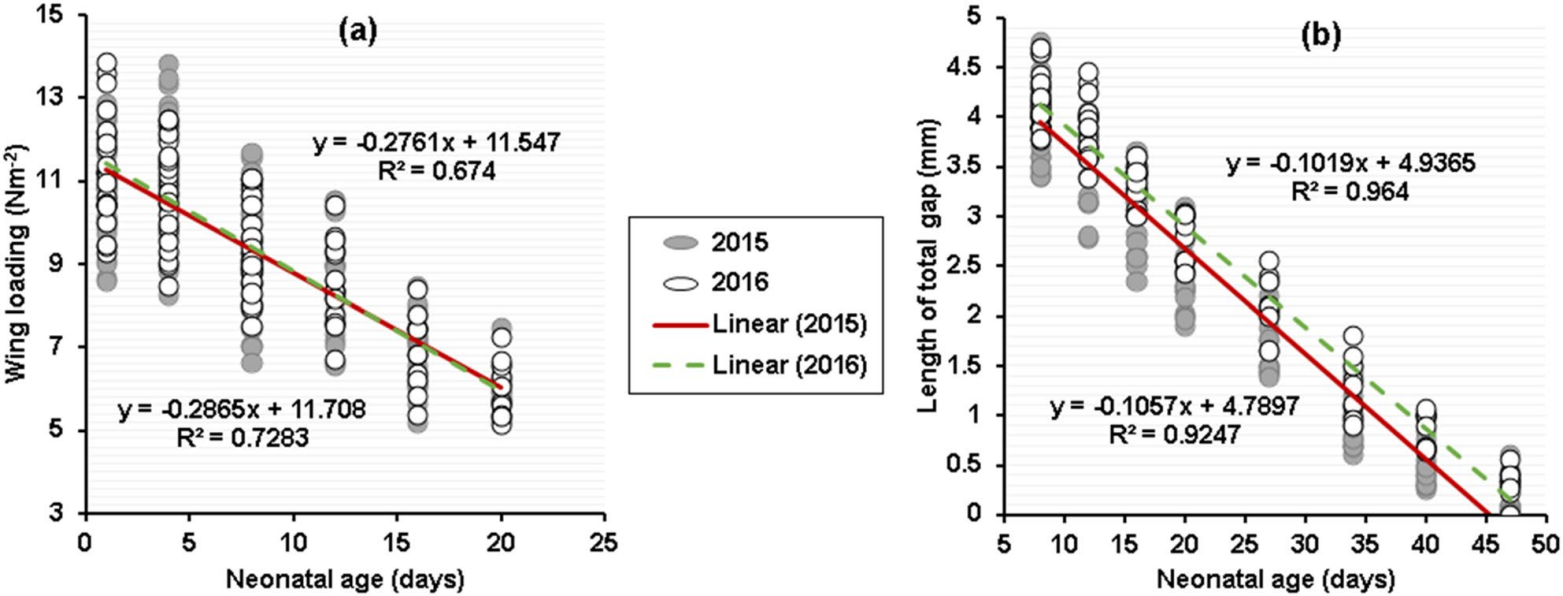
A comparison of age versus size in *Myotis* *emarginatus* in 2 years, based on the early linear portion of postnatal growth (wing loading, days 1–20, see^79^) and the latter linear phase of postnatal growth (length of total gap, days 8–47, see^79^). 2015 is represented by closed circles and a solid line and 2016 is represented by open circles and a dashed line. For both growth variables, the rate of changes did not differ between years (wing loading, GEE slope, P = 0.76; length of total gap, GEE slope, P = 0.18).

In both dry and wet years, 1-day-old pups had a high wing loading of 10.76 N m^−2^ (SD = 0.21, n = 28) and 11.28 N m^−2^ (SD = 0.27, n = 24), respectively. The wing loading decreased linearly until 20 days of age in both years, when they attained a mean wing loading of 5.96 N m^−2^ (2015, SD = 0.50, n = 20) and 5.99 N m^−2^ (2016, SD = 0.68, n = 10) which was equal to 91.27% and 87.31% of adults (Fig. 8a). For this growth derivative, tests for both equal intercepts (group term, P = 0.87) and parallelism (interaction term, P = 0.76) were not significant (Table 3). Similarly, for total epiphyseal gap length, in both years, tests for equal intercepts (group term) and parallelism (interaction term or growth rate) were not significantly different (GEE y-intercepts, P = 0.09; GEE slope, P = 0.18; Table 3). Thus, the growth rate lines start with same intercepts and were parallel (Fig. 8b).

## Discussion

### Reproductive phenology

The timing (i.e., phenology) of various key life cycle events, including emerging of female bats from a wintering site into a nursing cave, can have significant impacts on both newborn pups and their mothers by providing them with a place of appropriate climate conditions to help fertilize spermatozoa having been stored in female reproductive tracts in previous winter or autumn in the course of their mating with male bats in the wintering cave^80,81^. In our study, we surveyed whether a period of extremely moisture with low ambient temperature compared with ordinary dry and warm period could affect timing of the nursing congregation of female bats, synchrony and seasonality of parturition and postnatal growth of three species of bats that regularly occupy the Kerend cave each year^40,54^. Additionally, we investigated whether an extreme climate event could leave any impact on the reproducing bat species at population level through the change in growth and fitness or body condition index (body mass/forearm length) and community level (species composition and harmonic foraging) as a result of their being exposed to unfavorable environmental conditions.

There are multiple published studies aiming at uncovering the ecological and evolutionary impact of weather at the population or community level^1,11,42,81–83^. Variability in weather conditions has been known to influence survival and reproductive success in many animals including bats^2,44^. It has been reported that in temperate regions, inter-annual variation in weather tends to influence the timing of parturition in Chiroptran species by altering foraging conditions and food availability^47,52,84^. Length of torpor and gestation is likely to be connected with ambient temperature, as prolonged torpor allows parturition to be delayed until satisfactory conditions occur^51^. Similarly, availability of food seems to be a key factor determining when temperate insectivorous bats give birth. For example, parturition in lesser mouse-eared bats *Myotis* *blythii* is postponed in years when prey is uncommon^47^. Inter-year variation in precipitation has also been reported to trigger delays in postnatal growth and later parturition in Chiropteran species^37^.

Studies on interaction of food availability and ambient temperature have shown that reproduction in insectivorous bats is dependent on insect availability and is not delayed when there is a sufficient food despite low temperatures and high precipitation levels^48^. In temperate regions, female bats may benefit from warmer climates by bringing about earlier parturition and weaning of young bats. This offers more time for mating and feeding to store fat reserves in preparation for hibernation^11,42^. Similarly, earlier pregnancy and parturition may benefit juveniles by providing a longer growth period prior to the breeding season^11^.

High precipitation with associated lower ambient temperature in spring may cause reduction in foraging efficiency and a delay in parturition or even prevent the first-year breeding^2^. In an extreme case of premature parturition, juvenile mouse eared bats *Myotis* *myotis* were born 6 months earlier, suggesting that hibernation was abandoned due to warm and dry autumnal conditions^84^. However, by influencing reproductive phenology, specifically the timing of reassembly and cave departure, birth synchrony, the timing of parturition and the duration of gestation, the environmental conditions can have a disproportionate effect on breeding success in temperate insectivorous bats, but species differ in their relative sensitivity to climatic variation, possibly mediated by niche partitioning and differences in habitat use, reproductive requirement and foraging strategies^2,11,38,42,85^. The present study reported different responses to low temperature and high precipitation where the timing of parturition in *R*. *euryale* and *R*. *ferrumequinum* followed different patterns in dry and wet years, and there was not a significant difference in *M*. *emarginatus*. Similarly, although all three species showed individualistic shifts in timing of arrival to the nursing colony in response to high precipitation, *M*. *emarginatus* did not show significant differences.

### Birth synchrony and seasonality

Previous studies have demonstrated that phenology (repeated seasonal biological events) is a critical part of ecological relationships, and a primary indicator of species responses to climate change^15^. In a seasonal temperate zone, delayed fertilization, reduction in fetus development and timing of parturition could be affected by unfavorable climatic conditions in insectivorous bats^86–88^. Further surveys have revealed that under cool and wet conditions, delayed parturition and lactation by up to 1–2 weeks were evident in several vespertilionid bats^86^, along a delay of nearly 30 days in parturition dates and as well as a delay in birth synchrony in *Antrozous* *pallidus*^45^, *Myotis* *lucifugus*, *Myotis* *yumanensis* and *Myotis* *cilioabrum*^37^.

In the present study, birth synchrony in *R*. *euryale* exhibited different patterns in 2015 and 2016. In 2016, when births were less synchronous (births were spread over more days), total monthly precipitation from January to May was significantly more than similar data in 2015 (P < 0.05). Furthermore, mean monthly temperature during the prenatal growing month (May) and the postnatal growing periods (June–July) were significantly colder. As a result, these conditions may have induced the delayed parturition in 2016. The differences between dry and wet years for the Mediterranean horseshoe bat were very similar to those observed for *R*. *ferrumequinum*, which showed lower synchrony of parturition in 2016. *Myotis* *emarginatus* females gave birth to a single pup in June (with no precipitation) in both years. However, in this study, there was not a significant difference in timing of births in this species. It is not clear why such dissimilar responses to the different climate situations can be revealed by the co-occurring bat species. Some researchers have suggested that differences in reproductive behaviors may be potentially be due to species-specific feeding and reproductive strategies^42^, or population-specific climate sensitivity^89^. The Geoffroy’s bat gives birth 10–15 days after the beginning of birth by the other two sympatric species when the cave is warmer and is better prepared for postnatal development. Furthermore, *M*. *emarginatus* uses its nursery roost for a shorter period of time.

### Size at birth and instantaneous growth rate

**Figure 9:**
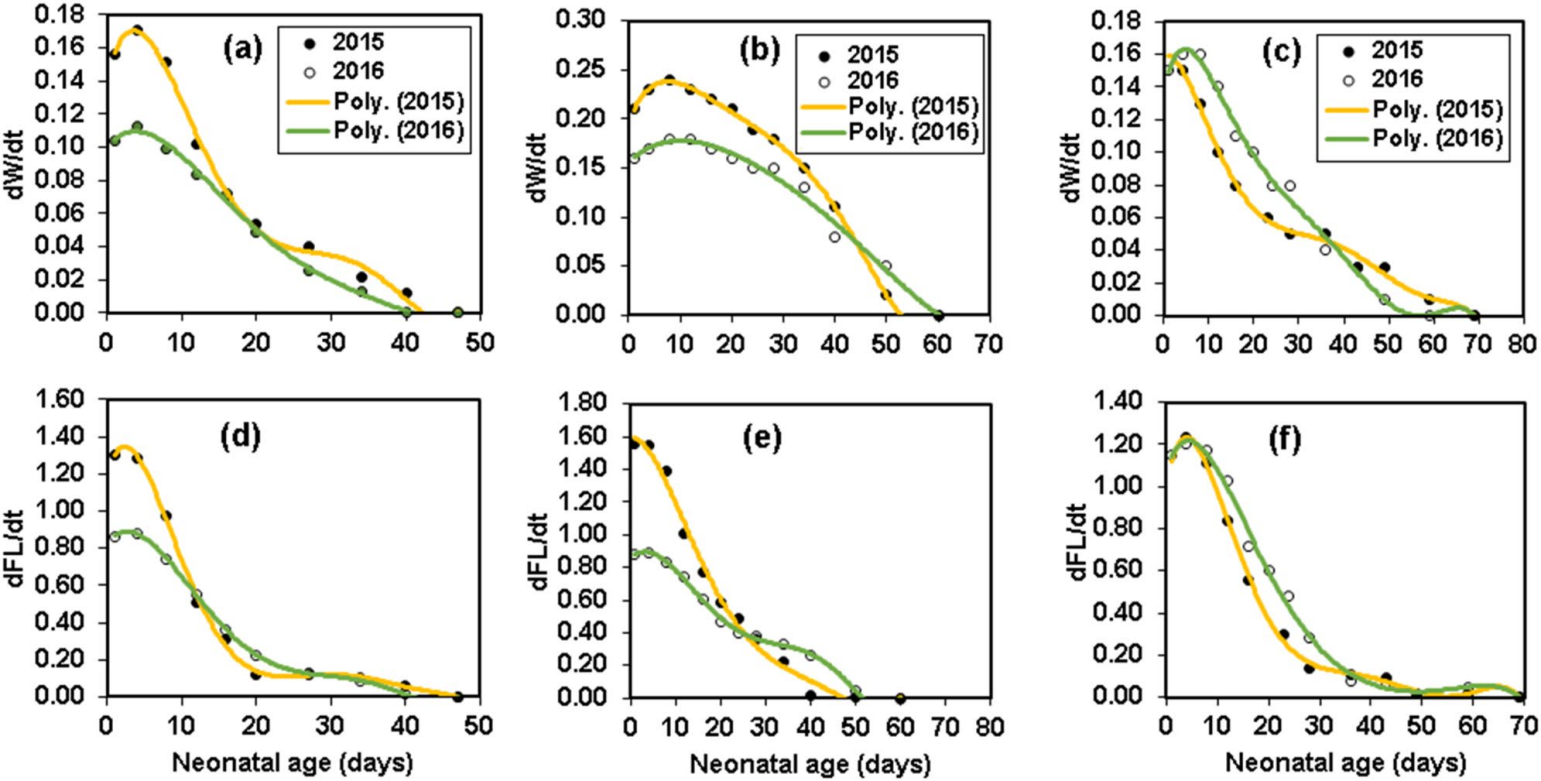
Spontaneous growth rate for body mass and forearm length in *Myotis* *emarginatus* (**a**,**d**, reproduced from^79^ for 2016 data), *Rhinolophus* *ferrumequinum* (**b**,**e**, reproduced from^40^) and *Rhinolophus* *euryale* (**c**,**f**) in dry (2015) and wet (2016) years.

Due to climatic conditions, it was predicted that bats born in 2016 would be smaller at birth compared with those born in 2015. Except for length of forearm in the Geoffroy’s bat (Fig. 7b), for *R*. *ferrumequinum* and *M*. *emarginatus*, the results contradicted this hypothesis and the body mass and wing parameters of juvenile bats were not different between 2015 and 2016 colonies, whereas for *R*. *euryale*, our findings are in line with our hypothesis (Fig. 5; Table 3). However, the differences were the greatest during the preflight period in growth rates in the three co-existing species. Multiple regression and generalized estimating-equation analyses indicated that in *R*. *ferrumequinum* and *M*. *emarginatus*, non-volante pups raised in the dry year growth at faster rates than those born in the wet year, as measured by BM and FL (Table 3). Conversely, for *R*. e*uryale*, during the first 4 weeks of postnatal growth, pups born in 2016 grew at faster rates than pups born in 2015, as measured by the same growth variables (Fig. 9). However, inter-annual differences in growth rates could be due to climatic conditions differences, although climate change may have unequal effects on different bat species. So that ecological factors in the year 2016 had a negative influence on the growth of *R*. *ferrumequinum* and *M*. *emarginatus*, while factors in this year favored growth in juvenile *R*. *euryale*.

### Different strategies amongst co-existing species

Our results demonstrated a series of associations between climatic conditions and life history traits (such as birth synchrony, timing of parturition and rate of postnatal growth) in sympatric bat populations. However, some responses of bats to the differing climate did not act uniformly among the three co-occurring bat species in the Kerend cave. The greater horseshoe bat gave birth earlier in 2015, whereas beginning of birth in the Mediterranean horseshoe bat and *M*. *emarginatus* was not affected by different dry (2015) and moist (2016) conditions. Nevertheless, for both horseshoe bats, the duration of the parturition varied between dry and wet years. A significant difference was found between years, with a longer period of parturition in 2016 (Mann–Whitney test: *P* < 0.05). However, for *M*. *emarginatus*, we found no significant differences in birth duration between 2 years (Mann–Whitney test: *P* < 0.05).

One way that bats cope with changes in their environment is altering their reproductive events. This strategy is common in temperate bat species, experiencing seasonal climate conditions. Kunz and Hood^90^ suggested that a long gestation period leading to parturition of large juveniles at birth may shift the risk caused by climatic conditions from juveniles to the mothers, who might be able to cope better with such situations. However, previous studies have confirmed that pups born earlier have higher survival rates compared with those born later, as Ransome^91^ showed for *R*. *ferrumequinum* in England and Frick et al.^2^ showed for *Myotis* *lucifugus* in Peterborough. However, climate changes may have unequal effects on different bat species due to differences in foraging, habitat and reproductive requirements. In some bat species such as *Myotis* *daubentonii*, due to their flexible hunting strategy, flight behaviour and hunting activity are not affected by air temperature or insect abundance^85^. Some studies indicate that the capture of insects at flight^92^ and their dependence on a food resource^42^, aerial-hawking species are highly sensitive to dynamics of prey abundance and weather conditions. In contrast, bats that forage on a variety of food sources, are not affected via insect densities and climatic changes^93^. In the present study, all three co-occurring species are aerial-hawking species, but with different home range and foraging strategies. For example, the greater horseshoe bat *R*. *ferrumequinum* is an aerial-hawking species^94^ that usually forage in short distance (up to 7 km) to their nursery roost^95^ and species has a highly specialized diet and is able to alter its foraging behaviour between seasons. *M*. *emarginatus* are adapted to hunting in multi stratified dense habitats and preys preferentially on spiders^70^.

### Effects of climate change on body condition index (BCI)

Bats are generally highly gregarious, forming large colonies inside caves known to have fairly constant temperature but can benefit from rapid torpor and keep neonates from freezing temperature during postnatal period. Bats’ exposure to fluctuated weather while most of time living inside stable environment results in natural selection of very diverse and adaptive reproductive cycle that enable the bat to survive in a changing climate^96^. However, there are few examples of research on the interaction between bats and differing climates. However, several authors have conducted simultaneous measurement of body mass/forearm length or body condition index (BCI) of neonates as metric to assess fat content, health and fitness of individuals and affected by nutritional status and habitat quality during the postnatal period^68,97–99^.

**Figure 10:**
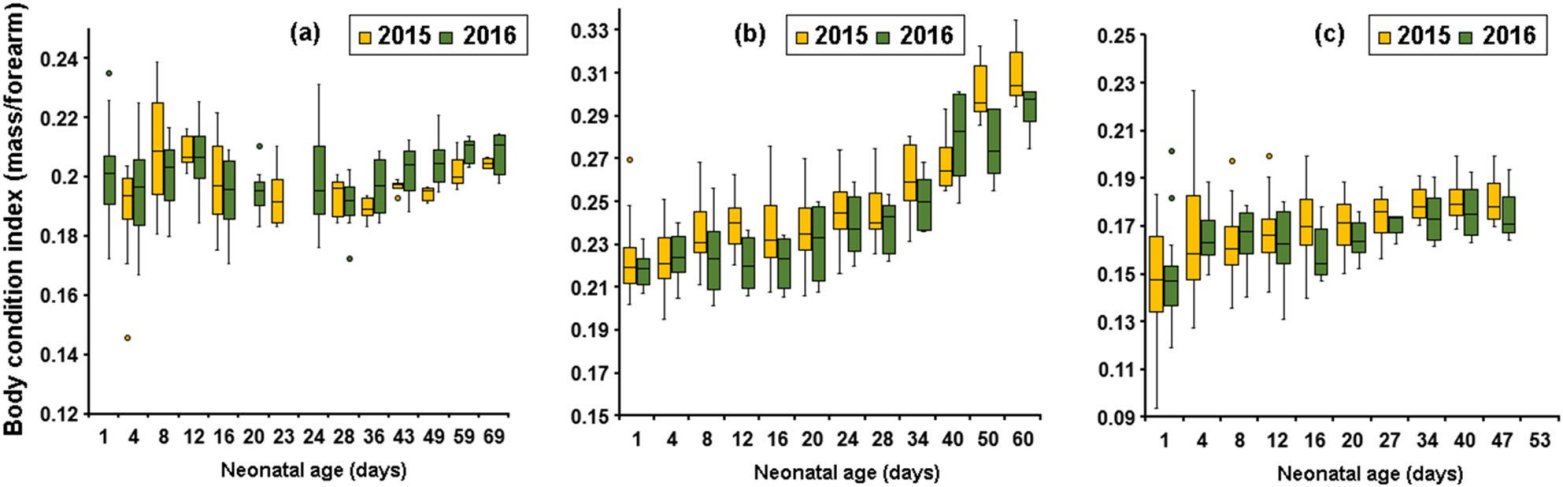
Body condition indices (BCI) defined as the ratio of body mass to forearm length explains the linear increase of body mass per unit of length of a bat during the postnatal period in *Rhinolophus* *euryale* (**a**), *Rhinolophus* *ferrumequinum* (**b**, see^40^), and *Myotis* *emarginatus* (**c**, see^79^). Boxes depict the 25th and 75th percentiles, lines within boxes mark the median, whiskers represent 95th and the 5th percentiles and dots indicate outliers.

In bat ontogeny studies, body mass and length of forearm are usually used as growth variables to monitor postnatal, behavioural and flight development. However, compared with body weight, forearm length grows faster and almost reaches adult dimensions during the postnatal period, whereas the asymptotic body mass of young bats is usually less than the adult mass. Lin et al.^100^ suggested that at a time when young bats are learning how to fly, low body mass is probably the reason for the rapid development of flight in young. After post-flight period, the higher quantitative increase of growth of body mass, compared with forearm length, explains the linear increase of body condition index until the end of postnatal period (Fig. 10). Our findings suggested that drought and warm conditions, which influence birth synchrony and postnatal growth rate, negatively affected the growth of *R*. *euryale* (Fig. 10a), while positively correlated with high fitness of *R*. *ferrumequinum* and *M*. *emarginatus*, expressed by BCI of the juvenile (Fig. 10b,c). Increased growth rates as a consequence of dry and warm weather conditions as found in the present study were also described for *R*. *ferrumequinum* in northern Bulgaria^73^. Although Dietz et al.^73^ showed that *R*. *ferrumequinum* individuals quickly reached adult dimensions in most external wing measurements in dry year (2003), while, *R*. *euryale* did not show a clear pattern^73^.

## Conclusions

Climate change is a global inevitable threat that has embarked on to put stress on biodiversity and ecosystem functioning. It is defined as increases in the hydrologic sequences and is expected to expand the frequency of extreme wet and dry events. The occurrence of intensified wet and dry periods has been shown to influence various aspects of reproduction cycles in large number of species at a different level of biological organization (species, population and community). In the present study, recruitment, seasonality and synchrony of birth and patterns of postnatal growth of neonates have been monitored in three co-occurring bat species in two different climates (dry and extremely wet) in individuals, populations and the bat community. In both years, assemblage of the three species remained unchanged, but timing of first arrival and last departure to/from the cave in both horseshoe bats expanded significantly during the wet year. *M*. *emarginatus* showed no significant differences in the pattern and timing of birth in the two dry and wet years. However, in all three species, timing of arrival and seasonality of birth and patterns of postnatal growth (body mass at birth, peak of postnatal growth, and rate of growth at early and late postnatal periods) have been affected in the two dry and wet years. Comparing various reproductive metrics among the co-existing bat species showed that the effects of climate on timing of behaviours related to reproduction are not uniform in these animals due to their different reproductive adaptations. Understanding the significance of variation in environmental conditions on life-history characteristics in the present study may help to predict how future climate changes could affect reproductive phenology.

## Data Availability

The datasets used and/or analysed during the current study available from the corresponding author on reasonable request.
